# Parenting concerns among patients with advanced early-onset lung cancer: a qualitative study based on the ABC-X model

**DOI:** 10.3389/fpsyg.2026.1930777

**Published:** 2026-09-24

**Authors:** Xiaoqian Zhou, Dan Yang, Liping Wang, Fen Wang

**Affiliations:** Department of Respiratory and Critical Care Medicine, Union Hospital, Tongji Medical College, Huazhong University of Science and Technology, Wuhan, China

**Keywords:** ABC-X model, early-onset lung cancer, parenting concerns, patients, qualitative research

## Abstract

**Introduction:**

To analyze parenting concerns of patients with advanced early-onset lung cancer based on the ABC-X model, thereby providing a reference for the development of targeted nursing intervention strategies in clinical practice.

**Methods:**

Using purposive sampling, 16 patients with advanced early-onset lung cancer who were raising minor children and were receiving treatment at a Grade A Level 3 hospital in Hubei, China, between August and October 2025, were selected for semi-structured interviews. Data analysis was conducted using Colaizzi’s 7-step method, and core themes were identified based on the ABC-X model.

**Results:**

Three main themes and seven subthemes were identified. Theme 1 was multiple parenting stresses and role conflicts (covering the stressor, Factor A), including dual physical and psychological constraints, learned helplessness amid parenting responsibilities, and economic conflict and role-adaptation imbalances. Theme 2 was insufficient family and social support resources (covering coping resources, Factor B), including weak social support and inadequate formal medical and welfare support. Theme 3 was negative cognitions and ineffective coping strategies (covering cognitive factors, Factor C), including catastrophic cognition and self-denial, as well as insufficient coping efficacy.

**Discussion:**

Parenting concerns among patients with advanced early-onset lung cancer were influenced by a combination of multiple stressors, insufficient support resources, and negative cognitive patterns. Nurses should implement targeted parenting support interventions based on the ABC-X model to strengthen family and social support, guide patients in positive cognitive reappraisal, alleviate parenting concerns, and improve their psychological adjustment and the quality of family care.

## 1 Introduction

According to the latest statistics from the International Agency for Research on Cancer (IARC), in 2022, global new cases of lung cancer were approximately 2.5 million, accounting for 12.4% of all new malignant tumor cases, and lung cancer deaths were approximately 1.8 million, accounting for 18.7% (Ferlay et al., 2024). Both the incidence and mortality of lung cancer ranked first among global malignant tumors (Ferlay et al., 2024). In China, the number of new lung cancer cases and lung cancer-related deaths also ranked first among all types of malignant tumors, and both have continued to increase in recent years (Diao et al., 2025). Early-onset lung cancer (EOLC) was defined as lung cancer occurring in adults under the age of 50. Its incidence was on the rise worldwide, with Asia accounting for approximately 76% of global EOLC cases (De Guzman, 2025). Within Asia, China accounted for nearly half of all new EOLC cases globally and more than one-third of all EOLC deaths (Cai et al., 2025). When such patients were diagnosed at the terminal stage of the disease, namely clinical stage IV A or IV B, the condition was defined as advanced EOLC (Asamura et al., 2023). Unlike older adults with lung cancer, patients with advanced EOLC were often in the prime of life. Beyond navigating illness-related stress stemming from diagnosis, treatment, and rehabilitation, this group bears heavy familial and social role obligations (Tu et al., 2025). Many of them fall into the “sandwich caregiver” predicament: while contending with a life-threatening malignant condition, they simultaneously take care of both minor children and ageing parents, a circumstance that further intensifies role strain and parenting-associated distress.

Existing studies have shown that young patients with cancer who have minor children are often caught in a severe conflict between the parental role and the patient role, and they face multiple parenting-related stressors, including a perceived decline in parenting ability, anxiety about role irreplaceability, and difficulties in decision-making regarding disclosure of the illness (Lai et al., 2025). Furthermore, the disease burden of lung cancer patients was transmitted through the family system, and the quality of the family environment significantly impacts patients’ mental health (Siminoff et al., 2010). However, for EOLC patients who were already in the advanced stages of the disease at the time of diagnosis, disease progression results in irreversible physical damage and an uncertain prognosis. The prolonged treatment process may cause severe physical discomfort such as fatigue, pain, and impaired immune function. It may also induce a range of negative emotions, including anxiety, depression, and fear (Karlsson et al., 2023; Zhang et al., 2023).

For patients with advanced EOLC who were also required to care for minor children, parenting concerns may arise as they faced their own survival crisis, particularly because the illness may interrupt their caregiving responsibilities. One study found that patients with lung cancer who were raising minor children experienced a heavier psychological burden (Li et al., 2025). Parenting concerns refer to the worry arising from an individual’s fear that his or her own illness may affect the growth and development of minor children (Rauch and Muriel, 2004). Previous studies have shown that parenting concerns can increase patients’ anxiety, depression, and other negative emotions, reduce their quality of life, and even influence their treatment decisions. Furthermore, parenting concerns can strain family relationships and have long-term negative effects on children’s psychological development (Tavares et al., 2020; Park et al., 2016; Inhestern et al., 2021). A deep understanding of the subjective experience of this concern is a key prerequisite for developing targeted support interventions and improving the overall quality of life for patients and their families.

The ABC-X model posits that the stress outcome (X) is the result of the comprehensive interaction of multiple factors, including the stressor (A), resources (B), and subjective cognition (C) (Schock-Giordano, 2013) and is further moderated by the individual’s available resources (B) and cognitive evaluation of the stressor (C). This theoretical model has been used to analyze the post-traumatic growth experiences of family caregivers of young and middle-aged patients with advanced cancer (Xiong et al., 2026). Parenting concerns in patients with advanced EOLC represent a psychological problem formed by the interaction of multiple factors, including disease-related stress, parenting responsibilities, and family support. The ABC-X model focuses on the pathways of family stress generation and allows for the differentiation of key variables such as objective stressful events, family coping resources, and individual cognitive appraisals, making it suitable for the purpose of this study, which is to explore the multiple causes and influencing factors of parenting concerns in patients with advanced EOLC. Therefore, this study aimed to analyze parenting concerns in patients with advanced EOLC within the theoretical framework of the ABC-X model, where A (stressor) refers to the parenting stress faced by patients with advanced EOLC, B (resources) refers to personal, family and social support resources, C (cognition) refers to the patient’s perception of parenting issues after a cancer diagnosis, and X (outcome) refers to parenting concerns. Using the ABC-X model as its theoretical framework and employing qualitative research methods, this study focuses on the experiences of parenting anxiety among advanced EOLC patients raising minor children, with the aim of providing a theoretical basis for developing a scientifically grounded, individualized parenting support care plan.

## 2 Methods

### 2.1 Design

A qualitative design was used in this study. The reporting of study procedures followed the recommendations outlined in the Consolidated Criteria for Reporting Qualitative Research (COREQ) checklist (Tong et al., 2007).

### 2.2 Study setting and participants

Using purposive sampling, the study recruited patients with advanced EOLC who were treated at a Grade A Level 3 hospital in central China between August and October 2025. Participants were selected based on sex, educational level, marital status, and the number of minor children they were raising to ensure diversity.

Inclusion criteria: (a) First-time histopathologically confirmed early-onset lung cancer, defined as a patient aged 18–49 years at diagnosis (De Guzman, 2025), with a clinical stage of IVA or IVB (Asamura et al., 2023), (b) Currently raising minor children (under 18 years of age), (c) Stable condition and able to communicate normally. Exclusion criteria: (a) Unstable mental status, (b) With other major comorbidities.

This study ultimately included 16 patients with advanced EOLC for interviews. We adopted concurrent data collection and iterative analysis. After completing each interview, two researchers independently transcribed the audio recording within 24 h and performed preliminary open coding of the transcript before recruiting and conducting the next participant interview. We continuously monitored for the emergence of new codes or themes. Recruitment was stopped when three consecutive interviews generated no newcodes or themes, which defined data saturation in this study. All participants took part in the entire process, with no dropouts midway. Participants were numbered P1–P16.

### 2.3 Interview guide

Based on the ABC-X model, we developed a preliminary interview guide through a literature review and internal discussions within the research team. We then conducted pilot interviews with two patients with advanced EOLC and finalized the interview guide based on the results of these pilot interviews. The interview guide was as follows: (1) A (Stressors): (a) Since becoming ill, at what moments have you found it difficult to fulfill your parental responsibilities? (b) From your personal, family, and work perspectives, what difficulties have you encountered in raising your children? (2) B (Resources): (a) What support do you feel you have received from your family, workplace, and the hospital regarding child-rearing? (b) What additional support would you like to receive? (3) C (Cognition): (a) How have your views on parenting changed before and after your illness? (b) How do you view the impact of your illness on your role as a father/mother and on your child’s future? (4) X (Outcomes): (a) How has your illness changed your family life and that of your child? The interview guide (Chinese original and English translation) is provided in Supplementary material 1.

### 2.4 Data collection

This study used a face-to-face semi-structured interview method. The researchers first screened patients who met the inclusion and exclusion criteria through medical records and nursing assessments, and conducted a preliminary communication with them during non-treatment time to explain the purpose and significance of the study, anonymity, and the confidentiality principle of audio recording. After the patients signed written informed consent forms, interviews were conducted by the female researcher who had received master’s level graduate training and formal instruction in qualitative methods. Appointments were made to conduct interviews in a quiet departmental meeting room to ensure a comfortable environment without external interference. All interviews were conducted during participants’ non-treatment periods (e.g., after infusion completion, between nursing procedures). During the interviews, techniques such as responding, restating, clarifying, and summarizing were comprehensively applied to encourage participants to express themselves fully, while their facial expressions and body language were recorded. Each interview lasted 30–45 min. The entire process was recorded using a fixed audio recording device, and the researchers simultaneously took field notes. No irrelevant personnel were present except for the two researchers and the participant. All interviews were conducted in Mandarin Chinese. Audio recordings were transcribed verbatim into written Mandarin transcripts before subsequent qualitative analysis.

### 2.5 Data analysis

Within 24 h of the interview, the audio recording was transcribed verbatim into a written transcript and imported into NVivo 15 software. This study employed Colaizzi’s seven-step method (Strahan and Brown, 2005) for in-depth data analysis, with the specific steps outlined below: (a) Read and comprehend the interview transcript, (b) Select statements of significant importance, (c) Code recurring meaningful themes, (d) Summarize the coded themes, (e) Write detailed, comprehensive descriptions, (f) Identify similar themes and distill overarching concepts, (g) Return to the interviewees for verification.

### 2.6 Ethical considerations

This study has been approved by the ethics committee of Union Hospital, Tongji Medical College, Huazhong University of Science and Technology (Ethics Approval Number: NTLYLL2023016). The researchers adhered to all ethical principles, including written informed consent, confidentiality, privacy protection, anonymization, and the participants’ right to withdraw from the study. All participants voluntarily signed the informed consent form and participated in this study.

### 2.7 Trustworthiness

This research adopted four criteria to improve the rigor of the study: credibility, transferability, reliability, and confirmability (Ahmed, 2024). Credibility was ensured through the following two measures. First, the interviewer (ZXQ) conducted at least two ward visits with each participant prior to the formal interviews, with a cumulative duration of no less than 2 h, to establish a relationship of trust. Second, triangulation was performed by two coders (ZXQ and WLP). Two researchers independently coded the interview transcripts, resolved discrepancies through discussion to reach a consensus, and returned the interview analysis results to the respondents for verification. Member-checking was performed for all 16 participants. For in-hospital participants, printed condensed thematic summaries were delivered face-to-face during ward visits. For discharged participants, thematic summaries were sent via WeChat. Participants could confirm, correct, or supplement interpretations. Feedback from participants was integrated to refine the thematic analysis. Transferability was enhanced through thorough documentation of the research context, sampling strategy, inclusion/exclusion criteria, and participant profiles. Readers can thereby evaluate whether these study findings are transferable and applicable to their own clinical and cultural settings. Reliability was ensured by maintaining a complete audit trail of all research stages and decisions. Confirmability was verified through peer review conducted by a clinical oncology nursing expert (WF).

## 3 Results

A total of 16 patients with advanced early-onset lung cancer were interviewed. Among them, 9 were female and 7 were male, with ages ranging from 33 to 47 years. Most participants were married (n = 12), and four were divorced. Participants’ educational attainment spanned from primary school to master’s degree. All participants had stage IVA/IVB disease and were receiving diverse anti-cancer treatments, including chemotherapy, targeted therapy, immunotherapy, and radiotherapy. Participants raised 1–3 minor children. Full demographic and clinical information is summarized in Table 1.

**Table 1:** Characteristics of participants.

ID	Sex	Age	Marital status	Educational level	Disease stage	Time of diagnosis	Treatment modalities	Number of minor children	Child’s gender and age
P1	Male	39	Married	Senior high school	IVA	1 year	①②③	1	Son:10 years old
P2	Male	38	Married	Senior high school	IVB	6 months	①②④	1	Daughter:9 years old
P3	Female	47	Married	Senior high school	IVB	2 years and 7 months	①②③	1	Son:16 years old
P4	Male	42	Divorced	Master’s degree	IVB	2 years and 5 months	②③	1	Daughter:15 years old
P5	Female	33	Married	Bachelor’s degree	IVB	1 year and 6 months	①②	1	Son:7 years old
P6	Female	38	Divorced	Junior college	IVA	3 years and 1 months	①②③	2	Daughter:8 years old Son:12 years old
P7	Female	40	Married	Junior high school	IVB	2 years	①③④	1	Son:12 years old
P8	Female	43	Married	Primary school	IVB	1 year and 1 months	②③	1	Son:15 years old
P9	Male	37	Married	Junior college	IVB	8 months	①②③	3	Daughter:13 years old Daughter:5 years old son:3 years old
P10	Male	43	Married	Bachelor’s degree	IVB	10 months	①③	1	Daughter:17 years old
P11	Female	40	Married	Bachelor’s degree	IVB	1 year and 3 months	②	1	Daughter:12 years old
P12	Male	39	Married	Junior college	IVA	1 year and 6 months	②③	1	Daughter:11 years old
P13	Female	47	Divorced	Senior high school	IVB	2 years	②③	1	Son:16 years old
P14	Male	37	Married	Bachelor’s degree	IVB	7 months	②④	2	Son:5 years old
P15	Female	40	Married	Bachelor’s degree	IVA	1 year and 7 months	①②③	2	Son:9 years old Daughter:15 years old
P16	Female	42	Divorced	Bachelor’s degree	IVB	9 months	②④	2	Daughter:16 years old Daughter:7 years old

Treatment modalities: ① Radiotherapy; ② Chemotherapy; ③ Targeted therapy; ④ Immunotherapy.

A total of three themes and seven subthemes were extracted from this study, constructing the formation mechanism of parenting concerns in patients with advanced EOLC (Figure 1).

**Figure 1:**
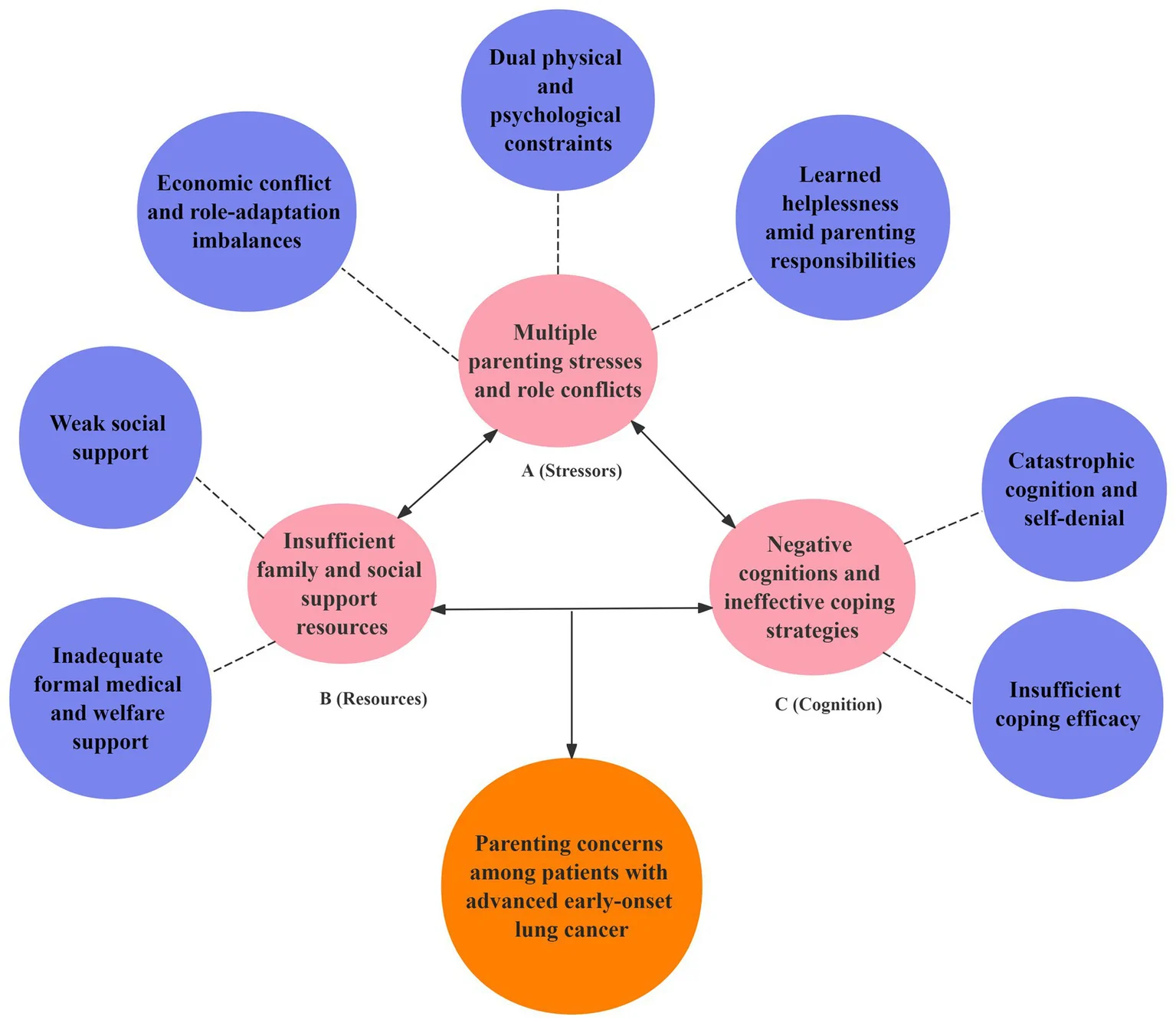
Formation mechanism of parenting concerns in patients with advanced EOLC.

### 3.1 Multiple parenting stresses and role conflict

#### 3.1.1 Dual physical and psychological constraints

From participants’ narratives, physical deterioration caused by advanced lung cancer and treatment side-effects limited patients’ capacity to perform parenting tasks. Meanwhile, many participants retained high pre-illness expectations for parental roles and struggled psychologically to accept reduced functional ability. We labelled this inductive theme dual physical and psychological constraints, capturing both embodied symptoms and intra-psychic burden reported by participants.

“*Since being diagnosed with cancer, my lung function has deteriorated significantly. If I walk too fast, I get a little short of breath. I used to take my son hiking and climbing in the mountains all the time, but I cannot do that anymore.*” *(P1)*

“*The chemotherapy made me vomit severely. I have no energy every day and cannot take care of my child.” (P6)*

Some patients remained attached to the role of an omnipotent parent as before the illness, struggled to accept the reality of their limited energy and abilities, and developed negative emotions. Persistent negative psychological states made it difficult for them to adjust their previous parenting expectations and practices, resulting in dual physical and psychological constraints.

“*I’m a very ambitious person, so I’ve always had high expectations for my child. I know I could lower my standards, but sometimes when I see my child’s less-than-ideal grades, I get very anxious and find it hard to stay calm about it.” (P13)*

*“I searched online and found that my type of cancer (small cell lung cancer) has the highest malignancy level, and I do not have much time left. If I were to pass away, it would undoubtedly have a devastating impact on my two children. But the more I think about it, the worse I feel physically.” (P16)*

#### 3.1.2 Learned helplessness amid parenting responsibilities

For some patients, the decline in physical functioning caused by disease progression led to a profound sense of helplessness when facing the responsibilities of parenting. Pessimistic expectations about their own prognosis and a lack of trust in others’ ability to provide care further intensify this sense of helplessness, ultimately causing them to adopt a passive, withdrawn, and resigned attitude toward their parenting role.

*“I do not know how much longer I’ll be able to be with my kids. I’ll just take it one day at a time. If I’m gone, their grandmother will take care of them. Their father has already started a new family, and I would not feel comfortable leaving them with him. My biggest concern right now is my two children, but there’s nothing I can do about it.” (P16)*

*“My physical condition has deteriorated after chemotherapy. Now their grandmother is looking after the children. Her health is only fair, and she is also rather careless in her daily tasks. I am not very comfortable with her taking care of them, but there is nothing I can do (the patient broke off, crying).” (P6)*

Although both P16 and P6 had to rely on their children’s grandmothers to undertake childcare responsibilities, participants demonstrated divergent appraisals toward grandparental care. For P16, grandmother-led care was accepted as an unavoidable fallback solution given her poor prognosis. By contrast, P6 expressed profound doubt regarding the grandmother’s capacity to adequately attend to children’s daily safety and needs. This contrast indicates that delegation of childcare to older family members does not inherently resolve patients’ parenting concerns, even when practical care arrangements are in place.

Furthermore, due to the high cost of treatment, patients with advanced EOLC often faced the difficulty of depleting their family savings, which affected their children’s educational investment and daily living resources, often leading to profound feelings of guilt.

*“I am ill and cannot earn money to support the family. I can only ask my daughter to focus on her schoolwork for now. I really cannot afford to continue her previous extracurricular activities, and I do not know how to change the current situation.” (P2)*

Meanwhile, due to the need for frequent and prolonged hospitalization, patients were unable to accompany their children regularly, leading to feelings of self-blame and helplessness.

*“My husband and I come from Henan every month for chemotherapy and check-ups. We are away from home for several days each time. It feels terrible. I feel I have let my two children down, but there is nothing I can do. I cannot change this.” (P15)*

Taken together, participants’ narratives of learned helplessness amid parenting responsibilities coalesced around three interconnected layers of experience. First, participants experienced helplessness when they were forced to hand over childcare to family members such as grandmothers, with persistent worries regarding care quality. Second, loss of work capacity and treatment-related financial hardship left participants feeling powerless to satisfy children’s material and educational demands. Third, frequent hospital admissions and physical deterioration interrupted routine hands-on parenting tasks. Across these three domains, participants perceived situations as uncontrollable, yielding passive, resigned psychological states, which we conceptualized as learned helplessness within this study.

#### 3.1.3 Economic conflict and role-adaptation imbalances

In the interviews, several patients mentioned the economic burden associated with lung cancer treatment. The high cost of treatment placed significant financial strain on both patients and their families, representing a considerable challenge faced by patients.

*“The cost of medical treatment is too high. When I was receiving treatment in Wuhan, the targeted therapy I took cost 15,000 yuan a month. Later, my doctor switched me to ensartinib, which costs over 5,000 yuan a month. It’s still expensive, and I’ve had to cut back a lot on expenses for my child.” (P8)*

*“My son is about to go to university. He is an art student and wants to continue studying fine arts, which will be very expensive. Money is needed everywhere.” (P3)*

As middle-aged individuals who played key roles in both society and the family, patients with advanced EOLC generally bore the triple responsibility of raising children, supporting aging parents, and advancing their careers. After being diagnosed with lung cancer, they were forced to take on the additional role of being a patient. Under the pressure of multiple roles, their parenting anxiety was further intensified.

*“I’m still working, and sometimes I have to take leave to go to the hospital for treatment. I spend less time and energy on my child than before.” (P5)*

*“Sometimes after a full day of work, I want to help my child with homework, but my body just cannot keep up like before. I just want to lie down and rest.” (P7)*

These accounts illustrated that advanced EOLC patients face heavy financial burdens from treatment, which compete with resources required for their children’s education and upbringing. As middle-aged caregivers, these patients juggle work and parenting while taking on the new role of a cancer patient, leading to role conflict. Physical fatigue and time shortages caused by illness reduce their capacity to perform parental duties, exacerbating multiple parenting stresses.

### 3.2 Insufficient family and social support resources

#### 3.2.1 Weak social support

The interviews revealed that some patients had been their children’s primary caregivers before falling ill, but due to the sudden onset of their illness, they were forced to rely on temporary outside support, such as short-term assistance from neighbors. In some families, although the children could be entrusted to their grandparents, the elders were often able to meet only their basic daily needs and found it difficult to address the children’s psychological and emotional needs.

*“My husband works away from home, so I take care of our son by myself. Sometimes when I go to the hospital for chemotherapy, he’s home alone, so I let him go out and buy his own food.” (P7)*

*“When my husband and I come to Wuhan for chemotherapy, we always ask our neighbor to help look after our daughter for a few days.” (P11)*

*“At home, my parents take care of the children, but they can only manage their basic needs like food and clothing. They have no way of attending to the children’s psychological well-being.” (P15)*

For young and middle-aged patients with lung cancer who were divorced, the absence of a spouse to share the burden often led to greater difficulties in raising their children.

*“Her mother and I divorced several years ago. My daughter has been living with me ever since. Now that I’m ill, naturally I cannot take care of her as well as before. To be honest, as a father, I fall short of a mother in many details, and now, given the situation, the family cannot really help much either.” (P4)*

*“After my divorce, I did not find another partner. I have always raised my child alone. It has been very hard, and now that I am ill, it is even more difficult.” (P13)*

#### 3.2.2 Inadequate formal medical and welfare support

Participants perceived that healthcare institutions prioritized physical disease treatment while neglecting family- and parenting-related psychosocial needs.

*“In the hospital, they only focus on treating the disease. No one has ever asked me about the difficulties I have in raising my children. Sometimes I actually wish the doctors and nurses could help me think about how to explain my illness to my children*.” *(P6)*

In addition, some respondents noted that the scope of coverage under the medical insurance system for serious and malignant diseases was limited. Even with medical insurance, many targeted therapies, immunotherapy drugs, certain tests, specialize outpatient services, and medications purchased outside of hospitals required out-of-pocket payments or involve high copayments.

*“The reimbursement rate for medical treatment outside Wuhan is very low. Each hospitalization saves little money, placing a significant financial burden on the family.” (P1)*

*“Many medications are out-of-pocket and not reimbursed. For example, genetic testing costs several 1000 yuan, which I have to pay myself. Sometimes, just a few injections to boost white blood cells can easily cost 10–20,000 yuan.” (P3)*

### 3.3 Negative cognitions and ineffective coping strategies

#### 3.3.1 Catastrophizing cognitions and self-denial

For patients with advanced EOLC, what they carry includes the responsibility of parenting, their sense of self-worth, and hopes for future planning. The physical and mental exhaustion and uncertainty brought about by lung cancer and its treatment profoundly impact patients’ perception of their own value, leading to deep self-negation and consequently fostering internalized catastrophizing perceptions.

*“The moment I learned I had lung cancer, my world collapsed. My child is still so young. I suddenly felt that I had become a burden to my family.” (P9)*

*“My daughter will take the college entrance examination next year. At this critical moment, I have contracted this illness, affecting the whole family. I am not a qualified father.” (P10)*

*“Now I cannot even attend the school parent–child sports day. How can I ever be a good father in the future?” (P2)*

Patients with advanced EOLC shoulder parenting obligations and expectations for their children’s future, while the physical and mental toll of the disease undermines their self-worth. These patients develop catastrophizing cognitions and self-denial, viewing themselves as a family burden and blaming themselves for disrupting their children’s important life events. Their inability to participate in their children’s daily and milestone activities further reinforces negative self-evaluation of their parental competency.

#### 3.3.2 Insufficient coping efficacy

Interviews revealed that patients’ limited knowledge regarding cancer contributed to insufficient coping efficacy, manifesting as ineffective or irrelevant coping behaviors. This phenomenon was observed in some patients who perceived a lung cancer diagnosis as a family catastrophe and, without scientific basis, attributed it to genetic factors, believing it would negatively affect their children.

*“My wife and children used to tell me not to smoke, but I did not listen. Now, it serves me right—getting lung cancer at such a young age.” (P9)*

*“I do not smoke, and no one in my family smokes. I think it’s a genetic problem. This will surely have a bad effect on my child in the future.” (P14)*

Some participants also indicated that, due to a lack of professional support and communication strategies, they were uncertain how to appropriately discuss their illness with their children. In the face of cancer, their coping strategies were inadequate.

*“Sometimes I want to talk with her about topics related to lung cancer, but she is still so young. I do not know how to tell her.” (P12)*

*“I do not know what to do either. I do not say anything, and neither does my family. We’re just scraping by like this.” (P14)*

Limited cancer-related knowledge reduces patients’ coping efficacy and leads to unscientific causal explanations for lung cancer. Many patients hold unfounded beliefs that their disease stems from genetics or personal misdeeds, generating worries about potential adverse impacts on their children. Without professional guidance, patients struggle to communicate their illness to their offspring and adopt passive coping approaches when confronting cancer.

## 4 Discussion

The present study found that the sources of parenting concerns among patients with advanced EOLC were multifaceted, encompassing dual physical and psychological constraints, learned helplessness amid parenting responsibilities, and economic conflict and role-adaptation imbalances.

Previous research (Karlsson et al., 2025) has shown that, compared with other cancer types, patients with lung cancer experience more severe symptoms such as sudden-onset cough, wheezing, dyspnoea, and pain. The decline in cardiopulmonary function limits daily activities and role performance and readily triggers negative emotions. Caught in this dual physical and psychological discomfort, patients developed deep concerns about whether they could continue to fulfil their parenting responsibilities, which in turn induced learned helplessness. Learned helplessness refers to a negative, passive psychological experience that develops after an individual has been exposed to uncontrollable adverse events over a prolonged period (Lindroth et al., 1994). As the disease progresses, patients with advanced EOLC worry not only about their own prognosis but also, under the pressure of the parental role, about the difficulties involved in raising their children. The irresolvable burden of responsibility and uncertainty contribute to persistent parenting concerns. Further exacerbating patients’ perception of stress are economic conflicts and imbalance in role adaptation.

One study found that (Inhestern et al., 2016a; Inhestern et al., 2016b) the level of parenting concerns among patients was positively correlated with the number of minor children. An analysis of the reasons suggested that a higher number of children increased child-rearing expenses. These expenses, combined with the costs of treating the patient’s own illness, added to the patient’s economic burden (Zheng et al., 2023), forcing patients to make difficult trade-offs with limited resources and leading to pronounced economic conflicts. At the same time, patients constantly adjust between the sick role and the parental role. Treatment requires them to prioritize their own health as a patient, whereas parenting responsibilities demand that they continue to perform parental duties (Zhao et al., 2024). When these two roles become difficult to balance, role adaptation imbalance ensues. The results of the present study also confirm that the sources of stress among patients with advanced EOLC include economic conflicts and the multidimensional adaptation to the roles of patient and parent. Therefore, clinical changes require multi-level stakeholders to act collaboratively. First, oncology nurses are well-positioned to routinely screen for parenting concerns during admission and outpatient encounters. Second, hospital-based multidisciplinary psycho-oncology teams should develop brief clinical tools and guidance for patients about illness-disclosure communication with minor children. Hospital administrators can support the integration of family-focused psychosocial services within routine cancer care pathways. At the community level, community healthcare providers may build hospital-community-family linkages to deliver temporary childcare support for vulnerable families. Finally, health policy-makers should consider targeted financial subsidy schemes for families affected by advanced EOLC to mitigate financial toxicity. Notably, the multiple parenting pressures and role conflicts faced by patients with advanced EOLC do not exist in isolation. The lack of family and social support resources is a key external factor exacerbating patient stress, which the present study confirms as an important trigger for the persistent accumulation of parenting pressures and unresolved role conflicts.

The present study showed that patients with advanced EOLC faced a lack of resources in terms of family support and social security. At the family resource level, the main issues were inadequate family care, a shortage of emotional support, and a lack of human resources. Research indicated (Inhestern et al., 2016a; Inhestern et al., 2016b; Jiao et al., 2025) that inadequate family care has comprehensive negative effects on minor children. Patients who lack family support experience greater feelings of helplessness when facing both their illness and their childcare responsibilities, thereby exacerbating their own parenting concerns. In the present study, divorced patients, who lacked a spouse to share the burden and assume childcare responsibilities alone, held more negative perceptions of their prognosis and their children’s future. A study (Inhestern et al., 2017) also showed that single cancer patients report higher levels of parenting concerns than married patients. Therefore, healthcare professionals should understand the family structure of patients with advanced EOLC. For married patients, they should emphasize the positive supportive role of the spouse and encourage families to assume childcare responsibilities as a unit. For divorced patients, they should conduct in-depth assessments of the level of social and family support and provide mental health education to such patients, thereby helping patients with advanced EOLC maintain a stable treatment mindset and alleviate parenting concerns.

In the present study, most participants reported a high out-of-pocket proportion for medications, a low reimbursement rate for transregional medical insurance, and difficulty accessing continuous professional parenting support. These findings were consistent with the results of a conceptual analysis of parenting concerns among parents with cancer (Li et al., 2024). Previous research has shown (Phillips et al., 2022) that the level of parenting concerns among cancer patients is significantly negatively correlated with the level of social support they receive. When patients have insufficient access to social support resources, their level of parenting concerns increases. At the governmental and societal levels, dedicated financial subsidies should be provided to patients with advanced EOLC, and investment in childcare support services for families affected by cancer should be increased to effectively alleviate the dual burden of illness and childcare. Long-term insufficiency of family and social support resources prevents patients from effectively resolving the negative experiences arising from childcare pressures and role conflicts. Sustained stress and helplessness further distort patients’ cognitive patterns, thereby fostering negative cognitions and ultimately leading to ineffective coping behaviors.

The results of the present study showed that patients with advanced EOLC were prone to negative cognitions under the burden of intense parenting anxiety, which in turn led to ineffective coping. This finding was consistent with previous research (Kuswanto et al., 2020). From the perspective of the ABC-X model, cognitive factors play an important role in the formation of parenting concerns among patients with advanced EOLC. Patients’ attitudes toward and understanding of their lung cancer diagnosis and post-cancer parenting issues influence their emotional responses and behavioral coping strategies. In the present study, some participants believed that their sick role imposed multidimensional burdens on the family system, making it difficult for them to assume childcare responsibilities and provide financial support for the family. Consequently, they experienced negative feelings towards their self-worth and developed internalized catastrophizing perceptions. This finding is consistent with the study by He et al. (2024). Furthermore, as the present study focused on parenting concerns, the participants’ responses tended to reflect negative attitudes. The uncertainty associated with the prognosis of lung cancer may further exacerbate the catastrophizing perceptions of individual participants. The theoretical model of post-traumatic growth posits that (Tedeschi and Calhoun, 1996) individuals undergo multiple growth pathways after experiencing major traumatic events. A favorable social environment and family support can help patients develop positive stress-related behaviors and serve as a protective buffer against negative emotions. This suggests that healthcare professionals should assist patients in reconstructing their perception of the parenting role, affirming their parenting value and emphasizing the value of emotional companionship. At the same time, patients should be encouraged to actively seek support from family and friends when their own role function is limited, or they experience a loss of role identity, and to identify substitute figures who can share some of the parenting responsibilities, thereby alleviating role strain.

In summary, parenting concerns among patients with advanced EOLC are not caused by a single factor but rather constitute a negative cyclical mechanism formed by the mutual influence and interplay of multidimensional factors, including internal stress, external resources, and individual cognition. Multiple parenting pressures and role conflicts are the root causes of patients’ parenting concerns, providing stressful conditions for the generation of negative emotions and the emergence of maladaptive behaviors. The dual deficiency of family and social support resources further exacerbates the negative impact of role conflicts and parenting pressures, depriving patients of avenues for external assistance and emotional buffering, and leading to continuously escalating stress. On this basis, the negative cognitions and ineffective coping patterns that develop in patients are the result of the combined effects of excessive stress burden and lack of external resources. Furthermore, catastrophizing cognitions and insufficient coping efficacy can, in turn, aggravate difficulties in role adaptation, causing patients to become disconnected from social and family support systems and resulting in persistent and worsening parenting concerns. The mutual influence of these three factors explains the mechanisms by which parenting concerns among patients with advanced EOLC emerge, develop, and persist.

### 4.1 Limitations

The present study has several limitations. First, participants were recruited solely from one tertiary hospital in central China. Therefore, the transferability of these findings to other geographic regions, different healthcare systems, or distinct cultural contexts requires further qualitative inquiry. Second, the study included only the patients themselves and did not encompass the perspectives of family caregivers such as spouses or children, consequently, it may not fully capture the family system characteristics of parenting concerns. Third, data were collected through one-time interviews at a single time-point. Therefore, this study cannot illuminate the dynamic, evolving trajectory of parenting concerns as illness status changes and disease progresses over time.

### 4.2 Strengths

This study has several notable strengths. First, this is one of few qualitative investigations specifically focusing on parenting concerns among patients with advanced early-onset lung cancer. Second, this study was theoretically driven by the ABC-X family-stress model, which facilitates systematic interpretation of multi-layered interacting factors underlying parenting concerns rather than purely descriptive reporting. Third, we recruited a diverse sample with variation in sex, marital status, educational level, and the number and age of minor children, capturing heterogeneous lived experiences. The findings generate actionable implications for family-centered oncology nursing practice.

### 4.3 Future research directions

Several avenues merit future research. First, future multi-site qualitative studies recruiting participants from diverse geographic and healthcare settings can further test the transferability of our findings. Second, future research should incorporate multi-perspective data collection by interviewing spouses, caregivers, and minor children themselves, to capture family-system perspectives beyond only patient self-report. Third, longitudinal qualitative research with repeated serial interviews can track how parenting concerns evolve dynamically across disease progression phases. Fourth, mixed-methods studies can further quantify the prevalence and correlates of parenting concerns within this patient subgroup.

## 5 Conclusion

Based on the ABC-X model, the study found that patients with advanced EOLC experience multidimensional parenting stress and face challenges, including a systemic lack of support and ineffective coping dominated by negative cognitions. These findings suggest that healthcare professionals should attend to the multidimensional sources of stress in patients with advanced EOLC, value the family support effect of patients’ spouses, and provide professional parenting support interventions to guide patients in actively exploring and reconstructing self-meaning, thereby alleviating patients’ parenting concerns.

## Data Availability

The raw data supporting the conclusions of this article will be made available by the authors, without undue reservation.
