# Supplementary material for: Parenting concerns among patients with advanced early-onset lung cancer: a qualitative study based on the ABC-X model
Source: Front Psychol. 2026 Sep 24;17:1930777. doi: 10.3389/fpsyg.2026.1930777 (PMC13645533; doi:10.3389/fpsyg.2026.1930777)
Supplement: Supplementary file 1 [file Supplementary_file_1.doc]

**访谈提纲（中文版）**

（1）A（压力源）：①患病后，哪些时刻让您觉得难以履行作为父母的职责？②从您个人、家庭及工作的角度谈谈在育儿方面有哪些困难？
（2）B（资源）：①您觉得家庭、单位及医院在育儿方面给了您哪些支持？②您还希望有哪些方面的支持？
（3）C（认知）：①您觉得患病前后对于养育问题有哪些不同的看法？②您如何看待患病对您作为父亲/母亲角色，以及对孩子未来的影响？
（4）X（结果）：①生病后给您和孩子的家庭生活带来了怎样的改变？

Interview guide (English Version)

The interview guide was as follows:

(1) A (Stressors): (a) Since becoming ill, at what moments have you found it difficult to fulfill your parental responsibilities? (b) From your personal, family, and work perspectives, what difficulties have you encountered in raising your children?
(2) B (Resources): (a) What support do you feel you have received from your family, workplace, and the hospital regarding child-rearing? (b) What additional support would you like to receive?
(3) C (Cognition): (a) How have your views on parenting changed before and after your illness? (b) How do you view the impact of your illness on your role as a father/mother and on your child’s future?
(4) X (Outcomes): (a) How has your illness changed your family life and that of your child?
